# Transcriptomic Analysis Reveals Candidate Genes Responsive to *Sclerotinia scleroterum* and Cloning of the *Ss*-Inducible Chitinase Genes in *Morus laevigata*

**DOI:** 10.3390/ijms21218358

**Published:** 2020-11-07

**Authors:** Huanhuan Jiang, Xiaoyun Jin, Xiaofeng Shi, Yufei Xue, Jiayi Jiang, Chenglong Yuan, Youjie Du, Xiaodan Liu, Ruifang Xie, Xuemei Liu, Lejing Li, Lijuan Wei, Chunxing Zhang, Liangjing Tong, Yourong Chai

**Affiliations:** 1 College of Agronomy and Biotechnology, Southwest University, Chongqing 400715, China; jh8469259@email.swu.edu.cn (H.J.); jxy0606@163.com (X.J.); kamiyahiko@163.com (X.S.); xyf710@swu.edu.cn (Y.X.); jjy326@email.swu.edu.cn (J.J.); ycl416@email.swu.edu.cn (C.Y.); ad1994@email.swu.edu.cn (Y.D.); liu1500@email.swu.edu.cn (X.L.); xrf2019@email.swu.edu.cn (R.X.); lxm804527660@email.swu.edu.cn (X.L.); lilejing123@email.swu.edu.cn (L.L.); lijuanwei@swu.edu.cn (L.W.); wjmandzcx13@163.com (C.Z.); tlj6793@163.com (L.T.); 2 Academy of Agricultural Sciences, Southwest University, Chongqing 400715, China; 3 Chongqing Engineering Research Center for Rapeseed, Southwest University, Chongqing 400715, China; 4 Chongqing Key Laboratory of Crop Quality Improvement, Southwest University, Chongqing 400715, China; 5 Engineering Research Center of South Upland Agriculture of Ministry of Education, Southwest University, Chongqing 400715, China

**Keywords:** *Sclerotinia sclerotiorum*, Sclerotinia stem rot, mulberry, *Morus laevigata*, chitinase, RNA-seq

## Abstract

*Sclerotinia sclerotiorum* (Ss) is a devastating fungal pathogen that causes Sclerotinia stem rot in rapeseed (*Brassica napus*), and is also detrimental to mulberry and many other crops. A wild mulberry germplasm, *Morus laevigata*, showed high resistance to *Ss*, but the molecular basis for the resistance is largely unknown. Here, the transcriptome response characteristics of *M. laevigata* to *Ss* infection were revealed by RNA-seq. A total of 833 differentially expressed genes (DEGs) were detected after the *Ss* inoculation in the leaf of *M. laevigata*. After the GO terms and KEGG pathways enrichment analyses, 42 resistance-related genes were selected as core candidates from the upregulated DEGs. Their expression patterns were detected in the roots, stems, leaves, flowers, and fruits of *M. laevigata*. Most of them (30/42) were specifically or mainly expressed in flowers, which was consistent with the fact that *Ss* mainly infects plants through floral organs, and indicated that Ss-resistance genes could be induced by pathogen inoculation on ectopic organs. After the *Ss* inoculation, these candidate genes were also induced in the two susceptible varieties of mulberry, but the responses of most of them were much slower with lower extents. Based on the expression patterns and functional annotation of the 42 candidate genes, we cloned the full-length gDNA and cDNA sequences of the *Ss*-inducible chitinase gene set (*MlChi* family). Phylogenetic tree construction, protein interaction network prediction, and gene expression analysis revealed their special roles in response to *Ss* infection. In prokaryotic expression, their protein products were all in the form of an inclusion body. Our results will help in the understanding of the molecular basis of *Ss*-resistance in *M. laevigata*, and the isolated *MlChi* genes are candidates for the improvement in plant *Ss*-resistance via biotechnology.

## 1. Introduction

*Sclerotinia sclerotiorum* (*Ss*) is a significant fungal pathogen that causes Sclerotinia stem rot (SSR) in rapeseed (*Brassica napus* L.) [1,2]. Except for the rapeseed, as a nonhost-specific, omnivorous, and necrotrophic plant pathogen, *Ss* can infect more than 400 plant species around the world, and result in a significant yield and quality reduction in many important crops [3,4,5]. In China, the incidence of SSR in the rapeseed growing areas is about 10% to 80%, and yield loss of diseased plants ranges from 10% to 73%. Therefore, it is urgent to breed *Ss*-resistant rapeseed varieties. However, there is currently a lack of highly *Ss*-resistant breeding germplasm in rapeseed. In addition, although some researchers have explored the interaction relationships between various crops and *Ss* in multiple aspects [6,7,8,9,10], the genetic and molecular basis of the interactions of *Ss* with its host crops is still poorly understood.

Mulberry (*Morus* sp.) is a kind of deciduous tree, the leaves of which can be used as food for rearing silkworm (*Bombyx mori*), and its fruits not only have a delicious taste but are also health-promoting because of the rich nutraceuticals such as anthocyanin [11,12]. In order to encourage the leaves growth that can feed silkworms, the mulberry plants need to be trimmed regularly, which makes it susceptible to pests and pathogens [13]. Similar to rapeseed, the mulberry plants are also susceptible to sclerotiose [14]. Mulberry fruit sclerotiniosis is also called white fruit disease, which includes mulberry sorosus hypertrophic sclerote disease, mulberry sorosus parvulling sclerote disease, and mulberry sorosus diminuting sclerote disease caused by *Ciboria shiraiana*, *Ciboria carunculoides*, and *Scleromitrula shiraiana*, respectively [14,15]. Once the mulberry is infected with the pathogen, the fruit turns gray and begins to rot, which seriously affects its economic value [14]. Recently, several researchers also isolated the *Ss* from the diseased mulberry fruit [16,17]. After artificial inoculation with the ascospore of *Ciboria shiraiana* in rapeseed, the infected plants showed typical characteristics of SSR. With the artificial inoculation of *Ss* in mulberry, the morphogenesis symptoms of the infected fruit were consistent with the mulberry sorosus hypertrophic sclerote disease [18]. These results indicate that the pathogen of mulberry sorosus hypertrophic sclerote disease and the *Ss* in rapeseed can cross-infect each other. Interestingly, the natural sclerotiose disease of mulberry is more frequently caused by the other three pathogens than by *Ss*, though it is fatal to rapeseed. That is to say, between the two hosts, *Ss* prefers rapeseed over mulberry, or mulberry is more resistant than rapeseed to *Ss* naturally. Different mulberry stocks vary distinctly in their susceptibility to sclerotiose pathogens. A wild germplasm of the mulberry, *Morus laevigata* (Himalyan mulberry or “Long-fruit mulberry”), showed resistance to *Ss*, which means that it can be used as a resistant germplasm resource [19,20]. Therefore, a better understanding of the resistance mechanism of mulberry, especially of *M. laevigata* to *Ss*, is not only useful for digging out the resistance-related genes, but also significant for breeding *Ss*-resistant rapeseed varieties via biotechnology. Though all four pathogens (*C. shiraiana*, *C. carunculoides*, *S. shiraiana*, and *S. sclerotiorum*) can infect mulberry, *S. sclerotiorum* is not the major type. In rapeseed, however, *S. sclerotiorum* is the major type of pathogen, while the first three are absolutely minor ones. Therefore, we assume that the exchange of the major resistance genes between mulberry and rapeseed should be helpful to each other to enhance resistance to white rot disease. As *M. laevigata* is the most *Ss*-resistant stock in the genus *Morus*, we believe that the major *Ss*-inducible resistance genes cloned from *M. laevigata* will be valuable for molecular breeding of the *Ss*-resistance of *Ss*-prevalent crops such as rapeseed, sunflower, and soybean, and might be effective in enhancing white rot disease of mulberry itself too.

Pathogen-associated molecular pattern (PAMP)-triggered immunity (PTI) and effector-triggered immunity (ETI) are the two main reaction processes of the plants’ innate immune systems against the infection of pathogens [21]. However, the steps for the host plant immune response against *Ss* remain unclear. Analysis of the genome sequences of *Ss* indicated that its genome contains a large number of genes that encode secretory effector proteins that may be involved in the interaction between *Ss* and the host [22]. Though recent studies have shown that certain types of secreted proteins are important pathogenic factors, only a few studies have actually identified a host receptor of the *Ss* effectors [23]. For example, the small secreted protein SsSSVP1 was secreted from the hyphae and then located in the cytoplasm of the host, which could interact with the QCR8 protein of the host, resulting in changes in the subcellular localization of QCR8, and induce significant plant cell death [24]. Another secreted protein SsCP1 could interact with the PR1 protein of the host in the apoplast to facilitate infection of *Ss*, and over-expression of *SsCP1* in *Nicotiana benthamiana* could significantly induce cell death, while over-expression of *PR1* enhanced the resistance to *Ss* [25]. These results indicate that the potential effectors play important roles in the host–pathogen interactions during the infection of *Ss* to plants.

Chitin is another kind of effector, which is the major component of the cell walls of fungi, but not in plants [26]. However, chitinase is widely present in plant cells. When the plants are infected by pathogens, the activities of chitinase increase rapidly. It can catalyze the hydrolysis of β-1,4 glycoside bond linkages of chitin polymers of the fungal cell wall into N-acetyl glucosamine oligomers and monomer components, resulting in cell wall degradation and death of pathogens [22,26]. Hence, chitinase is also considered as the pathogenesis-related (PR) proteins in plants and has been widely used in genetic improvement of the disease resistance of crops by genetic engineering [27,28]. In mulberry, twenty chitinase genes (*Mnchi1–Mnchi20*) were identified from the *M. notabilis* genome, and some of them were confirmed to respond to insect wounding and fungal infection [29]. Heterologous expression of mulberry latex chitinase gene *MaMLX-Q1* in *Arabidopsis* enhanced the defense against *Plutella xylostella* of the transgenic plants [30]. In rapeseed, co-expression of a defensin gene from *Raphanus sativus* and a chimeric chitinase gene *chit42* from *Trichoderma atroviride* enhanced the resistance against SSR [31]. Additionally, co-expression of the *chit42* gene mentioned above and a polygalacturonase-inhibiting protein 2 gene (*PG1P2*) from *Phaseolusvulgaris* also showed increased resistance to SSR in transgenic rapeseed [32]. Therefore, the isolation of *Ss*-inducible chitinase genes from *Ss*-resistant mulberry cultivars has potential application value in breeding *Ss*-resistant rapeseed cultivars via genetic engineering.

To date, there is no report on the systemic identification of the *Ss*-responsive molecular mechanism and systemic cloning of *Ss*-responsive *Chi* genes from mulberry species. In this study, the transcriptomic changes of the *M. laevigata* leaves before and after *Ss*-inoculation were analyzed to identify the candidate genes in response to *Ss*. Then, the expression patterns of the selected candidate genes among different tissues and varieties of mulberry were analyzed to further verify their roles. Additionally, as functional annotation showed that chitinase genes constitute a core set of the *Ss*-responsive defensive genes, we isolated their full-length cDNAs and corresponding genomic sequences (gDNAs) from *M. laevigata*, and the molecular features of the genes and their encoded proteins were analyzed bioinformatically. Our results provide a basis for understanding the *Ss*-resistance mechanism of *M. laevigata*, and the *Ss*-inducible chitinase genes isolated in this study have potential application in the genetic improvement of *Ss*-resistance in crops such as rapeseed, sunflower, and soybean, which are highly susceptible to *Ss*.

## 2. Results

### 2.1. RNA-seq and de Novo Transcriptome Assembly

**Figure 1:**
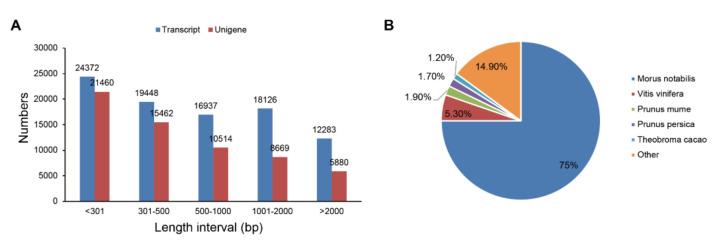
De novo transcriptome assembly of *M. laevigata*. (**A**) The length distribution of the *M. laevigata* transcripts and unigenes; (**B**) species distribution of the unigene BLAST results against the Nr database with the cutoff E-value of 10^−5^.

Two RNA-seq libraries (ML_SS0 and ML_SS1) were constructed in this study. Then, they were subjected to paired-end RNA sequencing using the Illumina HiSeq2500 platform, and on average, 25.98 million and 25.02 million clean reads were obtained from the two libraries, respectively (Table S1). Due to the lack of information about the *M. laevigata* genome, these clean reads were de novo assembled using the Trinity software [33]. Finally, a total of 91,166 transcripts and 61,985 unigenes were assembled, and the mean length of the unigenes was 784 bp and the N50 was 1487 bp (Table S1, Figure 1A).

### 2.2. Functional Annotation of the Unigenes

All of the unigenes were annotated using the BLAST algorithm in seven public databases with the E-value of 10^−5^. The results showed that 27,991 (45.15%), 14,341 (23.13%), 7258 (11.7%), 18,506 (29.85%), 18,463 (29.78%), 19,030 (30.7%), and 9779 (15.77%) unigenes were annotated in Nr, Nt, KEGG, Swiss-Prot, PFAM, GO, and KOG databases, respectively (Table 1). A total of 3804 unigenes were annotated in all of the seven databases, and 30,334 (48.93%) unigenes were annotated in at least one database. The unigenes of *M. laevigata* showed the best matches with *M. notabilis* (75.0%), followed by *Vitis vinifera* (5.3%), *Prunus mume* (1.9%), *Prunus persica* (1.7%), and *Theobroma cacao* (1.2%) (Figure 1B).

### 2.3. Analysis of Differentially Expressed Genes

**Figure 2:**
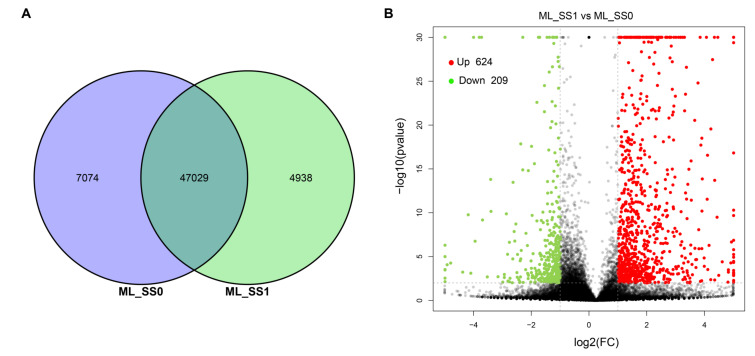
Screening of differentially expressed genes (DEGs). (**A**) Venn chart of co-expressed genes between the samples. (**B**) Numbers of up- and down-regulated DEGs in the infected leaves of *M. laevigata*.

**Figure 3:**
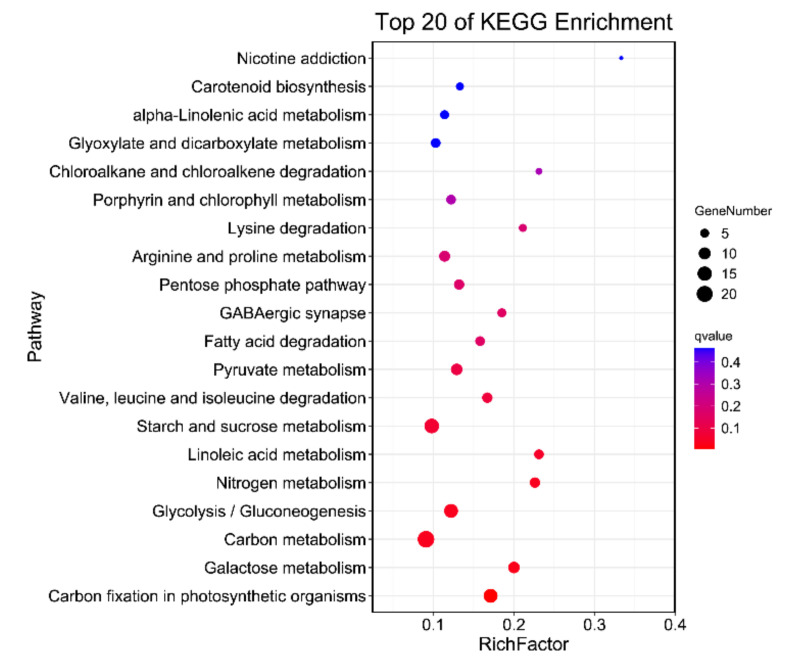
The top 20 statistics of KEGG pathway enrichment for the DEGs. The Rich factor is the ratio of DEGs in this pathway term to all the number of genes annotated in this pathway term.

By comparing the two libraries, a total of 47,029 common unigenes were identified in both of them (Figure 2A). The expression data of each gene were normalized using FPKM, and a total of 833 differentially expressed genes (DEGs) (624 upregulated and 209 downregulated in ML_SS1) were detected by the DESeq method with the threshold of adjusted *p*-value < 0.05 and |log2 (fold change)| ≥ 1 (Figure 2B, Table S2). Then, the DEGs were enriched in the GO and KEGG database. For the GO enrichment, the majority of the DEGs were enriched in the biological process (BP) and molecular function (MF) GO categories. The GO terms oxidation-reduction process (GO:0055114), single-organism metabolic process (GO:0044710), and metabolic process (GO:0008152) were mostly enriched in the BP category, and oxidoreductase activity (GO:0016491), endopeptidase inhibitor activity (GO:0004866), and endopeptidase regulator activity (GO:0061135) were the most significantly enriched GO terms in the MF category (Table S3). For the KEGG analysis, the 833 DEGs were enriched in 217 KEGG pathways (Table S4), and the 20 pathways with the highest enrichment are shown in Figure 3. Among the top 20 pathways, carbon fixation in photosynthetic organisms, galactose metabolism, glycolysis/gluconeogenesis, nitrogen metabolism, and carbon metabolism were significantly enriched, which include 14, 9, 14, 7, and 22 DEGs, respectively.

### 2.4. Candidate Genes Screening

**Figure 4:**
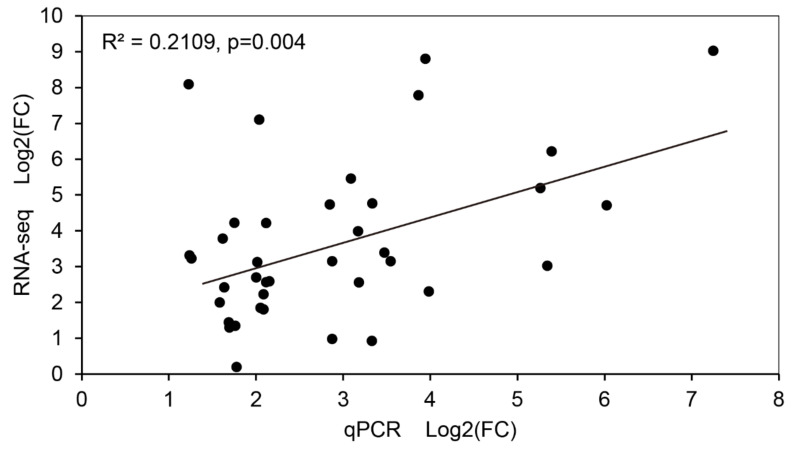
Correlation analysis of the gene expression fold change (FC) calculated from the quantitative real-time (qRT)-PCR and RNA-seq data. Pearson’s correlation (two tailed) was used for estimating p-values and r (n = 38) in SPSS 20.0.

According to the biological pathway description, several pathways that were closely related to disease resistance processes had been selected, such as the oxidation-reduction process, protein phosphorylation and proteolysis, metabolic process, and transcription factor and chitin metabolic process. Based on the expression levels of the DEGs in these biological pathways, a total of 42 upregulated DEGs were selected as the candidate genes in response to the infection of *S. sclerotiorum* (Table 2). These candidate genes were involved in endopeptidases, oxidoreductases, glucose metabolism, nitrogen metabolism, pathogenesis-related proteins, and transcription factors. Their expression levels were also validated using quantitative real-time (qRT)-PCR, which showed that the expression trend of 38 genes was consistent with that of RNA-seq, and only four genes (c30732_g1, c35494_g1, c40703_g1, and c45307_g1) had an inconsistent expression trend (Figure S2), which may be caused by the difference between the two detection platforms. For the above 38 genes, Pearson’s correlation was used to detect their expression correlation under the two platforms in SPSS software, and the results showed that the correlation between them reached a significant level (r = 0.459, n = 38, *p* = 0.004) (Figure 4).

### 2.5. Organ-Specificity of Transription Patterns of the Candidate Genes

**Figure 5:**
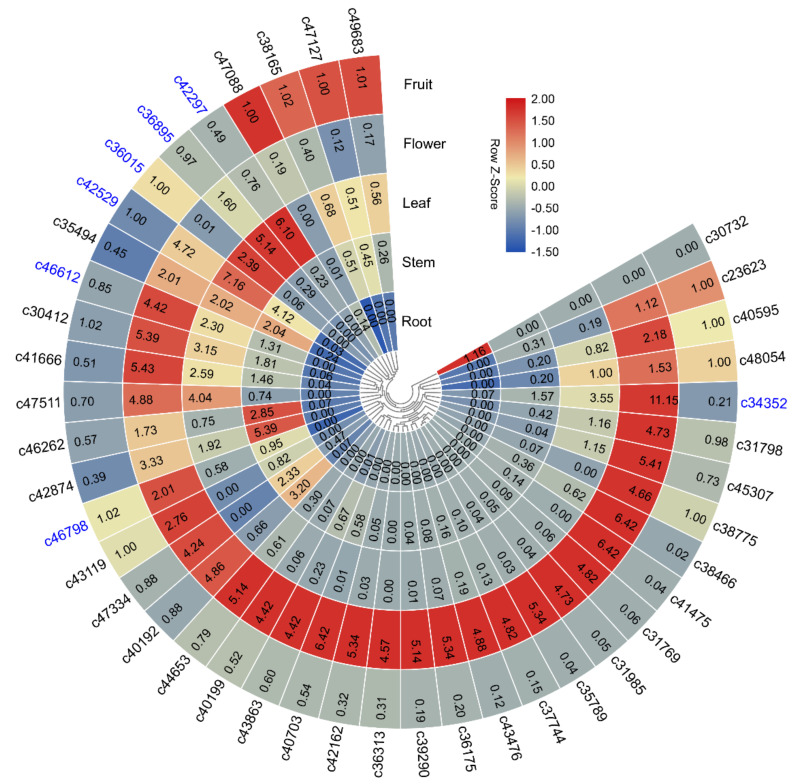
The expression patterns of the 42 candidate genes in different tissues of *M. laevigata*. The gene expression patterns were examined by qRT-PCR, and were visualized using the TBtools toolkit. The values in the heat map represent the relative expression levels of the genes. The expression patterns represented by the bar charts are shown in Figure S1.

The expression patterns of the 42 candidate genes were detected in the roots, stems, leaves, flowers, and fruits of the Ml using qRT-PCR, and most of them showed tissue-specific expression patterns (Figure 5). For example, *c30732* was specially expressed in roots, *c46262* and *c42874* were mainly expressed in stems, *c36895*, *c42297*, *c36015*, and *c42529* showed a high expression in leaves, and *c38165*, *c49683*, *c47088*, and *c47127* were predominantly expressed in fruits. Interestingly, the majority of the candidate genes (30/42) were specially or mainly expressed in flowers.

### 2.6. Stock-Diferred Expression and Ss-Inducibility Dynamics of the Candidate Genes

**Figure 6:**
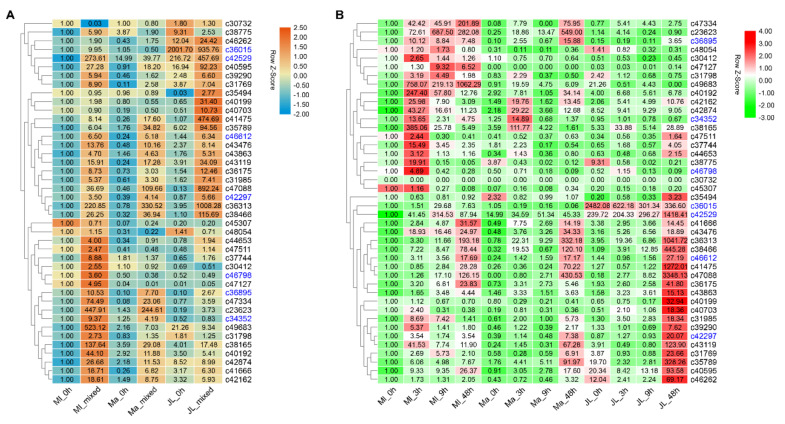
The expression patterns of the 42 candidate genes in different varieties of mulberry. (**A**) The expression patterns of the 42 candidate genes in the mixed samples at different stages after infection in different varieties. (**B**) The expression patterns of the 42 candidate genes at different stages after infection in different varieties. The gene expression analysis was examined by qRT-PCR, and all of the qRT-PCR values were expressed relative to the expression level of Ml_0h. The expression patterns represented by the bar charts are shown in Figures S2 and S3.

The expression patterns of the 42 candidate genes in response to the infection of *S. sclerotiorum* in different resistance stocks of mulberry were also detected using qRT-PCR. First, the expression levels of the candidate genes in the mixed samples of three cultivars at different periods after infection were analyzed (Figure 6A). As expected, almost all of them were upregulated in the three varieties after infection, but the extent to which they were expressed varied among resistance stocks. For example, *c45307, c23623, c34352, c47334, c36895, c43476, c38165, c40192, c37744, c49683, c30412, c46798, c47127, c47511, c44653, c31798, c42874, c41666*, and *c42162* were highly induced in Ml (high resistance to *Ss*), while *c46262, c40595, c47088, c40703, c40199, c41475, c35494, c35789, c36313, c38466, c42297,* and *c43119* were mainly induced in JL (partial resistance to *Ss*). However, their induction intensity in Ma (low resistance to *Ss*) was lower than those in Ml and JL.

Then, the expressions of the 42 candidate genes in different infection periods of the three resistance stocks were detected. As shown in Figure 6B, the majority genes were significantly induced in Ml with peak expression at 3 h after infection. More than half of them also showed certain inducibility in JL or in Ma, but with peaking expression much later than in Ml (e.g., 48 h after infection).

### 2.7. Cloning and Molecular Features of the Ss-Inducible Chitinase Genes from M. Laevigata

Among the 42 candidate genes, according to the functional annotation, unigenes *c36895*, *c42297*, *c46612*, *c46798*, *c34352*, *c36015*, and *c42529* were chitinase genes. Based on the homology with chitinase genes in other species, they were renamed as *MlChiIV, MlHEL, MlChiIA, MlChiV, MlChiIB, MlChiIC*, and *MlChiID*, respectively. Then, the full-length cDNA and corresponding gDNA sequences of the seven chitinase genes were isolated from *M. laevigata*. Finally, a total of fifteen homologous sequences were obtained and used for subsequent analysis. In sequence alignment, *MlChiIC1* was very similar to *MlChiIC2*, while *MlChiV2-1* and *MlChiV2-2* were very similar to *MlChiV1-2* and *MlChiV1-2*, respectively, which might be cloned from heterozygous allelic pairs. Due to the very high homology, their cloning primers were identical to each other, and could not be cloned by separate PCRs. In one-tube PCR gDNA-amplification for the highly homologous genes, the preferred amplification of one allele and suppressed amplification of another allele was a common phenomenon. We repeated the cloning process several times, and only gDNAs of *MlChiIC2*, *MlChiV1-2*, and *MlChiV1-2* were obtained, while gDNAs of *MlChiIC1*, *MlChiV2-1*, and *MlChiV2-2* were not obtained yet.

As shown in Table 3, except that *MlChiIC1*, *MlChiV2-1*, and *MlChiV2-2* did not obtain gDNA sequences, the length of gDNA and cDNA of the fifteen genes ranged from 1284 bp (*MlHEL2*) to 3603 bp (*MlChiV1-2*) and 753 bp (*MlHEL*) to 1332 bp (*MlChiIV2*), respectively. The numbers of amino acid (aa) residues of encoded proteins ranged from 144 (MlHEL) to 390 (MlChiV2), the relative molecular weight varied from 15.75 to 43.57 kDa, and their isoelectric point (*pI*) ranged from 4.62 to 6.84. The GRAVY of them varied from −0.42 to −0.20, showing that they were all hydrophilic proteins. Additionally, the signal peptide prediction showed that they all had signal peptides. Conserved domain analysis showed that MlChiIA, MlChiIB, and MlChiIV contained the Chitin_bind_1 domain (PF00187) and Glyco_hydro_19 domain (PF00182), MlChiV had the Glyco_hydro_18 domain (PF00704), MlChiID had the Chitin_bind_1 domain and Barwin domain (PF00967), while MlChiIC and MlHEL only contained the Glyco_hydro_19 domain and Barwin domain, respectively. Their subcellular location prediction showed that most of them were located in the vacuole except for MlChiV and MlHEL, which were located in the cell wall.

**Figure 7:**
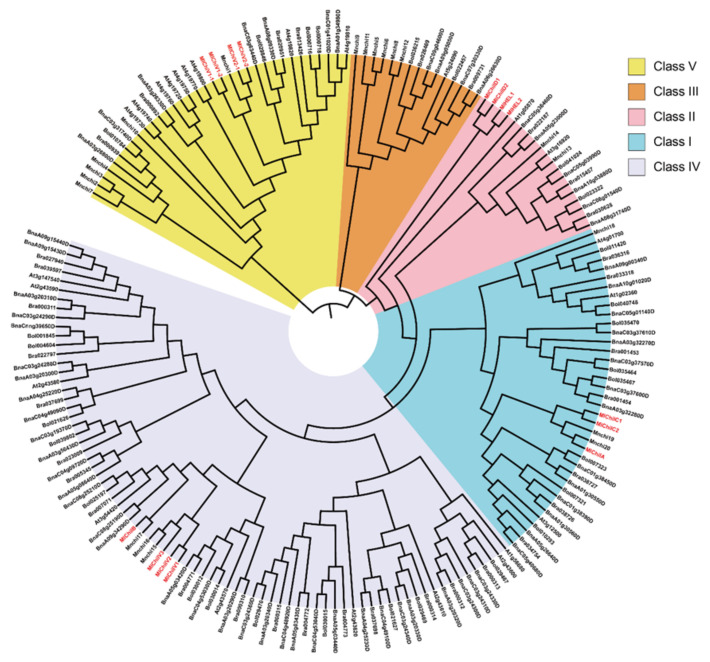
Phylogenetic relationship of the chitinases in different plants. The unrooted phylogenetic tree was constructed in MEGA6.0 using the NJ method with 1000 bootstrap replicates, and was visualized using the iTOL (https://itol.embl.de/) online tool.

The exon/intron analysis showed that except for *MlChiIA* and *MlChiIC*, which had two introns, all of the others only had one intron (Figure S4). The phylogenetic analysis of their full-length protein sequences showed that they could be divided into four groups (Figure S4), which was consistent with their exon/intron characteristics. Furthermore, the evolutionary relationships of the chitinases among different species were also explored. As shown in Figure 7, according to the evolutionary relationship of the chitinase gene family in mulberry [34] and Arabidopsis thaliana [35], these chitinases could be divided into five categories (Class I, II, III, IV, and V). The fifteen chitinases cloned in this study belong to Class I (*MlChiIA, MlChiIC1*, and *MlChiIC2*), Class II (*MlChiID1*, *MlChiID2*, *MlHEL1*, and *MlHEL2*), Class IV (*MlChiIB, MlChiIV1, MlChiIV2*, and *MlChiIV3*), and Class V (*MlChiV1-1, MlChiV1-2*, *MlChiV2-1*, and *MlChiV2-2*).

Then, the functional interactions of the nine chitinase proteins of *M. laevigata* were investigated in the STRING database with an *Arabidopsis* association model. The orthology analysis showed that *MlChiIV1*, *MlChiIV2*, *MlChiIV3*, and *MlChiIB* were homologous with the EP3 protein; *MlChiIA*, *MlChiIC1*, and *MlChiIC2* were homologous with the HCHIB protein; *MlChiV1-1*, *MlChiV1-2*, *MlChiV2-1*, and *MlChiV2-2* were homologous with the Chi protein; and *MlHEL1*, *MlHEL2*, *MlChiID1*, and *MlChiID2* were homologous with the PR4 protein of *Arabidopsis* (Table S5). Finally, a total of 66 interaction protein pairs were predicted in *Arabidopsis* (Table S6, Figure S5). *MlChiIA*/*MlChiIC* and *MlChiV* had more interaction proteins than *MlChiIV*/*MlChiIB* and *MlHEL*/*MlChiID*, and these four group proteins also showed varying degrees of interaction with each other (Figure S5). Additionally, GO enrichment analysis showed that the predicted interaction proteins were significantly enriched in defense response, immune response, response to external stimulus, systemic acquired resistance, chitin binding, and chitinase activity (Table S7).

### 2.8. Prokaryotic Expression of the Ss-Inducible Chitinase Genes from M. Laevigata

The prokaryotic expression of the fifteen chitinase proteins from *M. laevigata* was performed in *E. coli* BL21 (DE 3.0). As shown in Figure S6, all of the recombinant proteins could be detected by the induction with 0.5 mM IPTG, and the molecular weight of recombinant proteins was consistent with their theoretic values (Figure S6, Table 3). However, all the expressed proteins could only be detected in the precipitation fraction, not from the supernatant fraction, indicating that they are expressed as inclusion bodies. Considering that inclusion-body proteins were generally inactive, we did not carry out their purification and enzymatic activity detection.

## 3. Discussions

In this study, the transcriptome response characteristics of Ss-resistant mulberry stock *M. laevigata* after infection with *Ss* were revealed by RNA-seq. In total, 833 genes were differentially expressed after the infection. The DEGs analysis showed that most of them (74.9%) were upregulated, which means that they took part in the *M. laevigata*’s resistance to *Ss*. Consistent with the previous studies [7,10,15,36,37], these DEGs were mainly involved in the oxidation-reduction process and metabolic process, which indicated that the DEGs involved in these biological processes might play special roles in response to the *Ss* invasion. These results provide the possibility for us to explore *Ss*-resistance candidate genes.

Transcriptional changes are a way for plants to respond to pathogen invasion, especially the expression of some defense genes that can be significantly induced in this process [36]. Hence, we selected 42 resistance-related genes as the major candidate genes from the upregulated DEGs. Functional annotation showed that most of them encoded enzymes, such as transferases, reductases, oxidases, and kinases. These enzymes have been reported to play important roles in the defense responses against pathogen infection, including *Ss*, in different plants [10,38,39,40]. Some of them encode transcription factors, including MYB, NAC, ETR, TGA, and WRKY. Previous studies showed that these transcription factors played roles in the plant response to pathogen stress by controlling the synthesis of metabolites and affecting hormone signals, such as salicylic acid (SA), jasmonic acid (JA), and abscisic acid (ABA) signals [13,41,42]. For example, overexpression of *BnWRKY33* enhanced the resistance of the transgenic rapeseed to *Ss*, which might be due to activation of the SA- and JA-mediated defense responses [29]. Additionally, there are seven genes (*c36895*, *c42297*, *c46612*, *c46798*, *c34352*, *c36015*, and *c42529*) that encode chitinases among the candidate genes. Chitinases are often called pathogenesis-related proteins in plants, which play a dual role; they could inhibit pathogenic fungal growth by cell wall digestion, and could also release pathogen-borne elicitors to induce further defense reactions in the host plants [22,26]. They had been widely used to improve plant resistance to pathogens through biotechnology in various plants, such as tobacco [43], soybean [44], rapeseed [31,32], rice [45], and tea plant [46].

In order to further explore their roles in response to the infection of *Ss*, we analyzed their expression characteristics in different organs of *M. laevigata*. In plants, *Ss* mainly causes lesions (rot symptoms) in nonflower organs such as stem, silique, pod, and fruit, though the flower petal is the organ for easiest infection. To our surprise, most of them (30/42) are specific to or mainly expressed in flowers, which is consistent with the fact that *Ss* mainly infects plants through flower organs, especially the petals [3]. This phenomenon also indicates that *Ss*-resistance genes could be induced by pathogen inoculation on ectopic organs. This might be one of the reasons for *M. laevigata* to resist *Ss*. Then, we also compared and analyzed the expression characteristics of the 42 candidate genes in response to *Ss* infection in different mulberry resistance stocks. As all of them were quickly induced in resistant stock *M. laevigata*, most of these genes were also induced by *Ss* infection in the two stocks with partial or low resistance, but the response paces were much slower and the extents were generally lower. In *M. laevigata*, most of these genes were significantly upregulated at 3 h after infection. In low-resistance stock *M. alba* cv. Zhenzhubai (Ma) and partial-resistance stock *M. alba* × *M. atropurpurea* hybrid cv. Jialing 40 (JL), they were significantly upregulated at 48 h after inoculation. Generally speaking, the difference in response to pathogen invasion and resistance often depends on the difference in time of recognition and activation of their own defense system of plants [21]. If plants can quickly identify the pathogens, transmit signals, and initiate defensive responses, then they will show resistance in their interactions with pathogens. Otherwise, the plants will be affected. These phenomena had been observed in different studies [7,10], for example, Wu et al. found that the S-line rapeseed was more easily infected by *Ss* than the R-line, and the fold changes in many DEGs to the response to *Ss* inoculation were more dramatic in the R-line than in the S-line [2]. Therefore, the differences in the response of these candidate genes between *M. laevigata* and the two more-susceptible stocks are related to their resistance, which indicate that the differential expression of these candidate genes play an important role.

The genome of *Ss* contains a large number of different classes of virulence factors, including cell wall-degrading enzymes (CWDEs), signal cascade components, proteases and hydrolase, fungal nutrition and responding to environmental factors, reactive oxygen species (ROS) suppressors, and secreted proteins, which contributes to its wide range of infectivity and pathogenicity [23,47]. Therefore, it is necessary to fundamentally enhance its plant resistance. Among the 42 major candidates, chitinase genes of various types accounted for the largest group, so they were cloned and further studied. Cloning and functional investigation of the remaining candidate genes (e.g., the transcription factors) are also important for the resistance mechanism, which needs future study. Chitinase could degrade chitins, which are the primary structural components of the fungal cell walls, causing the cell walls’ degradation and death of pathogens [22,26]. In our study, for the seven *Ss*-inducible chitinase genes, we found that all of them were specially expressed in leaves or flowers and were apparently induced by *Ss* infection. Additionally, they also showed a more dramatic increase in expression levels at the early stage of infection in *M. laevigata* than in the two more-susceptible stocks (Figure 6). Wu and collaborators also found that the difference in the fold changes in defense-related genes, such as chitinases, were much more dramatic than the RLKs, MAPK, and WRKY genes between the R- and S-lines in response to *Ss* [2]. These results suggest that chitinase plays a primary role in response to *Ss* infection. Therefore, we then focused on the isolation and sequence analysis of these *Ss*-induced chitinase genes. Our results showed that the *Ss*-induced chitinase genes isolated from *M. laevigata* had the typical chitinase characteristics, such as relatively small molecular weight, fewer introns, and conserved domains, mainly located in the secretion pathway [48]. These also indicated the conservation of their functions. It has been proved that chitinase genes could respond to the infection of various pathogens in different plants [49,50], and their overexpression could enhance the disease resistance of plants [19,43]. Therefore, the full-length gDNA and cDNA of the *MlChi* genes were cloned in our study, which provided a basis for further functional research. The interaction between plants and pathogens was a complex process, which required a series of regulatory networks to play a role [21,28]. By using *Arabidopsis* homologous genes for protein interaction network prediction, we found that these MlChi proteins could be divided into four categories, and they interacted with many defense response proteins (Figure S5). These not only further indicated that they were involved in *Ss* response, but also revealed that they might participate in the *M. laevigata* response to *Ss* through interaction with other defense proteins.

We also tried prokaryotic expression of *Ss*-inducible *M. laevigata* chitinase genes, but they all yielded inclusion bodies. As chitinase proteins are generally hydrophilic low-weight stable molecules, their inclusion body phenomenon might not be caused by physical characters. However, they contain higher cysteine residue frequencies than common proteins, which means that their correct folding might be more complicated. Under common prokaryotic expression conditions, cysteine-rich proteins might be prone to misfolding and then form inclusion bodies [51]. As this research involves multiple types and a number of chitinases, their proper expression and enzymatic characterization are not a simple issue, which will be further studied systematically.

In conclusion, in this study, we uncovered the transcriptome characteristics of the response to *Ss* in *M. laevigata* and initially screened out 42 candidate genes that might play central roles in response to *Ss* infection. These candidate genes not only had tissue-specific expression, but also had differential expression in response to *Ss* infection between resistant and susceptible mulberry stocks. The cloning and expression analysis of the *Ss*-induced chitinase genes revealed their important functions in regulating the *M. laevigata* response to *Ss* infection. Our studies provided insights into the molecular basis of the resistance to *Ss* in *M. laevigata*, and the isolated *MlChi* genes could be potential candidate genes for the improving the *Ss* resistance of rapeseed and other crops through biotechnology.

## 4. Materials and Methods

### 4.1. Plant Materials and Treatments

In white rot disease, especially the Ss-resistance study, numerous papers have reported that in vitro inoculation on leaf or on stem is as effective as in vivo inoculation. In mulberry, white rot disease mainly causes heavy damage to fruits, the leaf can be infected, and light symptoms could be seen, while the stem of this tree can hardly be infected by *Ss*. As our purpose was to reveal the resistance mechanism and clone resistance genes, we chose mulberry leaf for in vitro inoculation. As mulberry trees are big and tall, and it is not easy to control and evenly treat under the open-air condition, we adopted the in vitro leaf inoculation method in this study. In early spring before the prevalence of natural *Ss* inoculation, young leaves were fetched for in vitro inoculation at room. Three mulberry varieties, including one wild resource *M. laevigata* (abbreviated as Ml, resistant to *Ss*) and two cultivated varieties, *M. alba* cv. Zhenzhubai (abbreviated as Ma, lowly resistant to *Ss*) and *M. alba* × *M. atropurpurea* hybrid cv. Jialing 40 (abbreviated as JL, partially resistant to *Ss*), were used in this study. All of them were planted in the mulberry orchard of Southwest University, Chongqing, China. The newly fully developed young leaves of the three varieties from the healthy plants were collected for *S. sclerotiorum* inoculation. The inoculated leaves were cultured in the dark at room temperature. After 0, 3, 9, and 48 h of inoculation, the leaf discs with a diameter of 2–3 cm around the inoculation point were collected as samples. Additionally, the leaves, flowers, stems, fruits, and roots of the Ml were also collected from the healthy plants as samples. Each sample had three biological replicates, and all of them were immediately frozen in liquid nitrogen and stored at −80 °C until further processing.

### 4.2. RNA Extraction, and cDNA Library Preparation and Sequencing

The total RNA of each sample was extracted using the EASYspin Plant RNA Kit (Biomed, Beijing, China) in accordance with the manufacturer’s instruction. The quality of the isolated RNA was detected by 1% agarose gel electrophoresis, and the concentration was checked with the Qubit 2.0 Flurometer (Life Technologies, CA, USA). Equal amounts of RNA from the 3, 9, and 48 h of *Ss*-inoculation of Ml were mixed together as the treatment sample (ML_SS1), and the 0 h sample of Ml was the CK sample (ML_SS0); they were used for the construction of RNA-sequencing libraries. The sequencing libraries were generated using NEBNext Ultra^™^ RNA Library Prep Kit for Illumina (NEB, MA, USA) following the manufacturer’s instructions, and index codes were added to attribute sequences to each sample. The RNA-seq was performed on the Illumina Hiseq 2000 platform with the Paired-end150 (PE150) strategy. The library construction and RNA-seq were performed by the commercial service of Novogene Co., Ltd. (Beijing, China).

### 4.3. Data Processing, and Transcriptome Assembly and Annotation

The raw reads of fastq format were first processed through in-house Perl scripts. Then, the clean reads were obtained by removing reads containing the adapter, the reads containing unknown bases (> 10%), and the low-quality reads (when the percentage of low-quality bases was over 50% in a read) from raw reads. At the same time, the Q20, Q30, GC-content, and sequence duplication level of the clean reads were calculated. Then, the clean reads were assembled using Trinity software [33] with min_kmer_cov set to 2 by default and all other parameters set to default to obtain the reference sequences, and the longest transcript of each gene was taken as the unigene for subsequent analysis. The gene function was annotated based on the following seven public databases: Nr (NCBI nonredundant protein sequences), Nt (NCBI nonredundant nucleotide sequences), Pfam (Protein family), KOG/COG (Clusters of Orthologous Groups of proteins), Swiss-Prot (A manually annotated and reviewed protein sequence database), KEGG (Kyoto Encyclopedia of Genes and Genomes database), and GO (Gene Ontology). The NCBI blast 2.2.28+ was used for functional annotation based on databases Nr (e-value = 1e–5), Nt (e-value = 1e–5), Swiss-Prot (e-value = 1e–5), and KOG/COG (e-value = 1e–3). The KAAS, HMMER3, and blast2go software were used for functional annotation based on databases KEGG (e-value = 1e–10), Pfam (e-value = 0.01) and GO (e-value = 1e–6), respectively. These steps were performed by the commercial service of Novogene Co., Ltd. (Beijing, China) according to its pipelines standards.

### 4.4. Differential Gene Expression, GO, and KEGG Enrichment Analysis

The expression levels of genes were estimated using FPKM (Fragment Per Kilobase of Exon Model Per Million Mapped Reads) [52] by RSEM software [53]. Differential expression analysis between the two samples was performed using the DESeq R package (1.10.1). The resulting *P* values were adjusted using the Benjamini and Hochberg’s approach for controlling for the false discovery rate (FDR). Genes with an adjusted *P*-value < 0.05 and fold change (FC) ≥ 2 (|log2 (fold change)| ≥ 1) identified by DESeq were assigned as significantly differential expression. The GO enrichment analysis was implemented using the GOseq R packages-based Wallenius noncentral hyper-geometric distribution, and the KEGG pathways enrichment analysis was performed using the KOBAS software.

### 4.5. Quantitative Real-Time PCR (qRT-PCR) Analysis

For the qRT-PCR analysis, the reverse transcription of mRNAs using the total RNA as the start sample was performed using the PrimeScript^TM^ RT Reagent Kit with gDNA Eraser (TaKaRa, Dalian, China) according to the manufacturer’s instruction. The specific primers of each gene were designed using Vector NTI Advance 11.51 and Primer Premier 6 software. The qRT-PCR reactions were carried out using FastStart Essential DNA Green Master (Roche Diagnostics GmbH, Mannheim, Germany) on a CFX96^TM^ Real-Time System (Bio-Rad, Irvine, CA, USA) with three technical replicates according to the manufacturer’s protocol. The *26SrRNA* was used as the internal reference gene and the relative expression level of each gene was calculated using the following formula: FC = 2^−^^∆∆CT^ [54]. The Bio-Rad CFX Manager 3.0 software was used for data analysis. The primers used in qRT-PCR analysis are listed in Table S8.

### 4.6. Cloning of cDNA and gDNA Sequences of Ss-Responsive Chitinase Genes from M. Laevigata

For the cloning of the chitinase genes from *M. laevigata*, the primers were designed based on comprehensive bioinformatics analysis, especially multi-alignment of the unigene sequences assembled in the *M. laevigata* transcriptome of this research, the *M. notabilis* genome references from the NCBI, and the in silico-cloned orthologous *Morus* EST and TSA tags from NCBI. The genomic DNA was isolated using the CTAB traditional method, and the cDNA of mixture-organ total RNA was obtained by reverse-transcription as described above. The PCR reactions were performed on the Veriti™ PCR instrument (Applied Biosystems, Foster City CA, USA). The amplification products were purified via an EasyPure Quick Gel Extraction Kit (Transgen Biotech, Beijing, China) and cloned into a pMD19-T vector for sequencing. The primers used in this study are listed in Table S8.

### 4.7. Sequence Characteristics, and Phylogenetic and Interaction Networks Analysis

For the bioinformatics analysis of the cloned chitinase genes of *M. laevigata*, the Conserved Domain Database (CDD, https://www.ncbi.nlm.nih.gov/Structure/cdd/cdd.shtml) was used to verify their protein sequences. The physico-chemical characteristics, including molecular weight (MW), theoretical isoelectric point (*pI*), and grand average of hydropathicity (GRAVY), were calculated using the online Program tool of ExPASy (http://www.expasy.org/tools/). The signal peptide (SP) and transmembrane domain were predicted using the online tool SignalP 4.1 Server (http://www.cbs.dtu.dk/services/SignalP/) and TMpred Server (https://embnet.vital-it.ch/software/TMPRED_form.html), respectively. Two online tools Plant-mPLoc (http://www.csbio.sjtu.edu.cn/bioinf/plant-multi/) and TargetP1.1 (http://www.cbs.dtu.dk/services/TargetP/) were used to predict the subcellular localization. The encoded amino acid sequences of the chitinase genes from different plants were aligned using Clustal X. Then, a phylogenetic tree based on the result of Clustal W protein alignments was constructed using the neighbor-joining (NJ) method in the software MEGA X with 1000 bootstrap replicates. Protein interaction network prediction was performed by STRING (https://string-db.org/) based on the homologous genes of *Arabidopsis thaliana* [55].

### 4.8. Prokaryotic Expression

The coding sequence of each gene with the signal peptide being removed was used as the template for primer design and vector construction. For *MlChiIA, MlChiIB, MlchiIC1/2, MlchiID1/2*, and *MlHEL1/2*, the fragment of each gene was cloned into the vector pET-28a (+), via the *Bam*HI + *Hin*dIII restriction endonuclease recognition sites. For *MlChiV1-1/2*, *MlChiV2-1/2*, and *MlChiIV1/2/3*, the vectors were constructed by *Bam*HI + *Not*I and *Sac*I + *Hin*dIII restriction enzymes, respectively. Then, the recombinant plasmid of each gene was transformed into *E. coli* strain BL21. The screened positive colonies of each gene were cultured in LB medium until the optical density (OD_600_ value reached 0.6 at 37 °C); then, they were induced overnight by IPTG with a final concentration of 0.5 mM at 28 °C. The recombinant *E. coli* cells were harvested by centrifugation from 5 mL culture medium after IPTG induction, and then they were resuspended with 1 mL Tris-NaCl (pH = 8.0). After centrifugation, the cells were resuspended again with 0.8 mL Tris-NaCl for ultrasonic crushing in an ice bath. The crushing mode was: Power, 300 W; ultrasonic, 1 s; interval, 3 s; and ultrasonic time, 5 min. After the samples were crushed, they were centrifuged at 4 °C and 12,000 rpm for 10 min to collect the supernatant and precipitation. Then, they were mixed with 5× loading buffer and were boiled for 10 min. The mixtures were centrifuged at 12,000 rpm for 5 min, and a total of 15 μL supernatant was analyzed by 12% SDS-PAGE. The primers used for vector construction are shown in Table S8.

### 4.9. Data Availability

The raw sequence data reported in this paper have been deposited in the Genome Sequence Archive [56] in National Genomics Data Center [57], Beijing Institute of Genomics (China National Center for Bioinformation), Chinese Academy of Sciences, under accession number CRA002928 that are publicly accessible at https://bigd.big.ac.cn/gsa. We are now submitting the sequences of the 42 unigenes and the cloned *Ss*-inducible chitinase genes to NCBI GenBank.

## Figures and Tables

**Table 1:** The statistical analysis of the annotated unigenes.

Databases	Annotated Unigenes	Percentage (%)
Nr	27,991	45.15
Nt	14,341	23.13
KEGG	7258	11.71
Swiss-Prot	18,506	29.85
Pfam	18,463	29.78
GO	19,030	30.70
KOG	9779	15.77
Annotated in all Databases	3804	6.13
Annotated in at least one Database	30,334	48.93
Total Unigenes	61,985	100.00

**Table 2:** The 42 candidate genes that were screened from the differentially expressed genes.

Seq_ID	log2 (FC)	Gene Annotation	BP Description
c49683_g1	7.25	Protein SRG1 [*Morus notabilis*]	oxidation
c30732_g1	5.04	Myb-related protein 305 [*Morus notabilis*]	reduction
c40595_g2	3.34	Cytochrome P450 82A3 [*Morus notabilis*]	process
c46262_g2	3.33	NAC domain-containing protein 8 [*Morus notabilis*]	
c38775_g1	3.18	Hyoscyamine 6-dioxygenase [*Morus notabilis*]	
c40192_g1	3.09	putative inactive poly [ADP-ribose] polymerase SRO5 [*Morus notabilis*]	
c35789_g1	2.15	putative linoleate 9S-lipoxygenase 5 [*Morus notabilis*]	
c38165_g1	2.04	Extracellular ribonuclease LE [*Morus notabilis*]	
c36175_g1	2.01	Myb-related protein 305 [*Morus notabilis*]	
c47127_g2	3.98	Calcium-dependent protein kinase 3 [*Morus notabilis*]	protein
c23623_g1	3.94	type II proteinase inhibitor [Potato]	phosphorylation
c40703_g1	3.65	indole-3-acetic acid amido synthetase [*Morus alba* var. multicaulis]	and proteolysis
c31769_g1	3.54	Salutaridinol 7-O-acetyltransferase [*Morus notabilis*]	
c43119_g1	3.17	Protein BONZAI 3 [*Morus notabilis*]	
c40199_g1	2.87	Protein kinase byr2 [*Morus notabilis*]	
c42162_g2	2.12	xyloglucanase inhibitor 3 [*Humulus lupulus*]	
c48054_g1	1.78	G-type lectin S-receptor-like serine/threonine-protein kinase RKS1 [*Morus notabilis*]	
c47511_g1	1.69	L-type lectin-domain containing receptor kinase VII.1 [*Morus notabilis*]	
c31798_g1	1.69	Cysteine proteinase inhibitor 5 [*Morus notabilis*]	
c31985_g1	1.64	Proteinase inhibitor [*Morus notabilis*]	
c36313_g1	3.87	Transcription factor MYB86 [*Arabidopsis thaliana*]	transcription
c37744_g1	2.87	Ethylene-responsive transcription factor [*Morus notabilis*]	factor
c42874_g1	2.85	Ethylene-responsive transcription factor [*Morus notabilis*]	
c39290_g1	2.12	Pathogenesis-related protein transcriptional activator PTI5 [*Morus notabilis*]	
c43863_g1	2.09	Transcription factor TGA1 [*Morus notabilis*]	
c45307_g1	1.83	putative WRKY transcription factor 33 [*Morus notabilis*]	
c30412_g1	1.76	Transcription factor MYB44 [*Arabidopsis thaliana*]	
c44653_g2	1.58	putative WRKY transcription factor 33 [*Morus notabilis*]	
c38466_g2	6.02	Zeatin O-glucosyltransferase [*Morus notabilis*]	metabolic
c47334_g1	5.39	putative cysteine desulfurase [*Morus notabilis*]	process
c35494_g1	5.38	Flavonol reductase [*Morus notabilis*]	
c41475_g1	5.34	Galactose oxidase [*Morus notabilis*]	
c47088_g3	5.26	Carbonic anhydrase 2 [*Morus notabilis*]	
c41666_g1	1.75	Glucan endo-1,3-beta-glucosidase, basic vacuolar isoform [*Morus notabilis*]	
c43476_g1	1.62	GDSL esterase/lipase 1 [*Morus notabilis*]	
**c36895_g1**	3.47	Endochitinase [*Morus notabilis*]	chitin metabolic process
**c42297_g2**	2.09	Pathogenesis-related protein P2 [*Morus notabilis*]	
**c46798_g2**	2.05	class V chitinase [*Morus alba*]	
**c46612_g1**	2	Endochitinase 1 [*Morus notabilis*]	
**c34352_g1**	1.26	Endochitinase [*Morus notabilis*]	
**c36015_g1**	1.24	class I chitinase, putative [*Ricinus communis*]	
**c42529_g1**	1.23	class I pathogenesis-related protein 4 [*Ficus pumila*]	

**Table 3:** Characteristics of the fifteen *MlChi* genes isolated from *M. laevigata* in this study.

Gene name	Seq_ID	gDNA	cDNA	ORF	AA	SP	MW (kDa)	*pI*	GRAVY	Domain	Subcellular localization
		(bp)	(bp)	(bp)							
*MlChiIA*	c46612	1997	1265	972	323	Y	34.95	6.84	−0.37	Chitin_bind_1 and Glyco_hydro_19	vacuole
*MlChiIB*	c34352	2167	1176	840	279	Y	30.39	4.63	−0.32	Chitin_bind_1 and Glyco_hydro_19	vacuole
*MlChiIC1*	c36015	N	1052	750	249	Y	27.36	5.57	−0.36	Glyco_hydro_19	vacuole
*MlChiIC2*	c36015	1548	1039	750	249	Y	27.73	5.10	−0.39	Glyco_hydro_19	vacuole
*MlChiID1*	c42529	1664	1052	633	210	Y	22.34	6.76	−0.31	Chitin_bind_1 and Barwin	vacuole
*MlChiID2*	c42529	1666	1054	636	211	Y	22.41	6.76	−0.29	Chitin_bind_1 and Barwin	vacuole
*MlHEL1*	c42297	1629	753	435	144	Y	15.78	6.79	−0.20	Barwin	cell wall
*MlHEL2*	c42297	1284	753	435	144	Y	15.75	6.04	−0.21	Barwin	cell wall
*MlChiV1-1*	c46798	3600	1307	1140	379	Y	42.15	4.96	−0.22	Glyco_hydro_18	cell wall
*MlChiV1-2*	c46798	3603	1307	1140	379	Y	41.98	4.88	−0.21	Glyco_hydro_18	cell wall
*MlChiV2-1*	c46798	N	1308	1173	390	Y	43.51	5.89	−0.23	Glyco_hydro_18	cell wall
*MlChiV2-2*	c46798	N	1308	1173	390	Y	43.57	6.14	−0.27	Glyco_hydro_18	cell wall
*MlChiIV1*	c36895	1847	1329	822	273	Y	29.48	4.66	−0.42	Chitin_bind_1 and Glyco_hydro_19	vacuole
*MlChiIV2*	c36895	1849	1332	822	273	Y	29.48	4.62	−-0.42	Chitin_bind_1 and Glyco_hydro_19	vacuole
*MlChiIV3*	c36895	1848	1331	822	273	Y	29.39	4.61	−0.38	Chitin_bind_1 and Glyco_hydro_19	vacuole

AA: Amino acid. SP: Signal peptide. MW: Molecular weight. *pI*: Isoelectric point. GRAVY: Grand average of hydropathicity.

## Supplementary Materials

Supplementary materials can be found at https://www.mdpi.com/1422-0067/21/21/8358/s1.

## Abbreviations

SsSSVP1	A small secreted virulence-related protein 1 in *Sclerotinia scleroterum*
QCR8	Cytochrome b-c1 complex, subunit 8 protein
SsCP1	Cerato-platanin protein 1 in *Sclerotinia scleroterum*
PR1	Pathogenesis-related protein 1
MaMLX-Q1	Latex chitinase protein 1 isolated from Mulberry variety Qiangsang 1 (Q1)
BLAST	Basic Local Alignment Search Tool
IPTG	Isopropyl beta-D-thiogalactopyranoside
