# Supplementary material for: Transcriptomic Analysis Reveals Candidate Genes Responsive to Sclerotinia scleroterum and Cloning of the Ss-Inducible Chitinase Genes in Morus laevigata
Source: Int J Mol Sci. 2020 Nov 7;21(21):8358. doi: 10.3390/ijms21218358 (PMC7664649; doi:10.3390/ijms21218358)
Supplement: Supplementary file 1 [file ijms-21-08358-s001.zip › Supplementary Table S1-3-8.docx]

**Table S1**. The quality statistics of the sequencing.

| Samples | Raw Reads | Clean reads | Clean bases | Error (%) | Q30 (%) | GC (%) |
| --- | --- | --- | --- | --- | --- | --- |
| ML_SS0_1 | 27,232,261 | 25,982,291 | 3.25G | 0.03 | 91.26 | 45.3 |
| ML_SS0_2 | 27,232,261 | 25,982,291 | 3.25G | 0.04 | 86.72 | 45.26 |
| ML_SS1_1 | 26,411,506 | 25,019,736 | 3.13G | 0.03 | 91.52 | 45.02 |
| ML_SS1_2 | 26,411,506 | 25,019,736 | 3.13G | 0.04 | 87.42 | 44.98 |
|  | Min Length | Mean Length | Median Length | Max Length | N50 | Total Nucleotides |
| Transcripts | 201 | 969 | 532 | 15,084 | 1721 | 88,327,898 |
| Unigenes | 201 | 784 | 395 | 15,084 | 1487 | 48,566,735 |

**Table S3.** The significantly enriched GO terms of the differentially expressed genes.

| GO terms | GO_accession | Description | DEGs | P-value |
| --- | --- | --- | --- | --- |
| biological_process | GO:0055114 | oxidation-reduction process | 133 | 6.25E-15 |
|  | GO:0044710 | single-organism metabolic process | 235 | 4.92E-12 |
|  | GO:0008152 | metabolic process | 448 | 4.02E-07 |
|  | GO:0005975 | carbohydrate metabolic process | 80 | 5.49E-06 |
|  | GO:0006040 | amino sugar metabolic process | 10 | 6.41E-05 |
|  | GO:0006066 | alcohol metabolic process | 13 | 0.000153 |
|  | GO:1901615 | organic hydroxy compound metabolic process | 13 | 0.000167 |
|  | GO:0044264 | cellular polysaccharide metabolic process | 34 | 0.000585 |
|  | GO:0019751 | polyol metabolic process | 11 | 0.000658 |
|  | GO:0009225 | nucleotide-sugar metabolic process | 11 | 0.000699 |
|  | GO:0006026 | aminoglycan catabolic process | 7 | 0.000844 |
|  | GO:0006073 | cellular glucan metabolic process | 20 | 0.000965 |
|  | GO:0044042 | glucan metabolic process | 20 | 0.000965 |
| cellular_component | GO:0048046 | apoplast | 5 | 0.000621 |
| molecular_function | GO:0016491 | oxidoreductase activity | 121 | 3.93E-11 |
|  | GO:0004866 | endopeptidase inhibitor activity | 12 | 6.47E-10 |
|  | GO:0061135 | endopeptidase regulator activity | 12 | 6.47E-10 |
|  | GO:0051213 | dioxygenase activity | 25 | 1.87E-09 |
|  | GO:0030414 | peptidase inhibitor activity | 12 | 2.74E-09 |
|  | GO:0061134 | peptidase regulator activity | 12 | 2.74E-09 |
|  | GO:0003824 | catalytic activity | 407 | 5.80E-09 |
|  | GO:0004553 | hydrolase activity, hydrolyzing O-glycosyl compounds | 40 | 7.06E-08 |
|  | GO:0016701 | oxidoreductase activity, acting on single donors with incorporation of molecular oxygen | 15 | 7.44E-08 |
|  | GO:0016705 | oxidoreductase activity, acting on paired donors, with incorporation or reduction of molecular oxygen | 38 | 2.57E-07 |
|  | GO:0016702 | oxidoreductase activity, acting on single donors with incorporation of molecular oxygen, incorporation of two atoms of oxygen | 12 | 4.44E-07 |
|  | GO:0016798 | hydrolase activity, acting on glycosyl bonds | 40 | 4.46E-07 |
|  | GO:0048037 | cofactor binding | 49 | 7.04E-07 |
|  | GO:0004857 | enzyme inhibitor activity | 16 | 3.12E-06 |
|  | GO:0050662 | coenzyme binding | 37 | 2.33E-05 |
|  | GO:0004427 | inorganic diphosphatase activity | 5 | 2.47E-05 |
|  | GO:0009678 | hydrogen-translocating pyrophosphatase activity | 4 | 6.30E-05 |
|  | GO:0016762 | xyloglucan:xyloglucosyl transferase activity | 5 | 0.000241 |
|  | GO:0016758 | transferase activity, transferring hexosyl groups | 29 | 0.000266 |
|  | GO:0004779 | sulfate adenylyltransferase activity | 3 | 0.000276 |
|  | GO:0004781 | sulfate adenylyltransferase (ATP) activity | 3 | 0.000276 |
|  | GO:0016706 | oxidoreductase activity, acting on paired donors, with incorporation or reduction of molecular oxygen, 2-oxoglutarate as one donor, and incorporation of one atom each of oxygen into both donors | 13 | 0.000328 |
|  | GO:0016614 | oxidoreductase activity, acting on CH-OH group of donors | 26 | 0.000394 |
|  | GO:0016757 | transferase activity, transferring glycosyl groups | 34 | 0.000662 |
|  | GO:0016616 | oxidoreductase activity, acting on the CH-OH group of donors, NAD or NADP as acceptor | 24 | 0.000817 |

**Table S8**. The primers used in this study.

| Seq ID | Primers | Primer sequence (5'-3') | Application |
| --- | --- | --- | --- |
| 26SrRNA | F | GTTACCACAGGGATAACTGGCTTG | qRT-PCR |
|  | R | CTAACCTGTCTCACGACGGTCTAA |  |
| c30732 | F | CATGTCCGACGTGTCAGGTA |  |
|  | R | CTGTTGCTGGAAGAACCGTAGA |  |
| c40595 | F | ACGAGTCCACAGAAGACTGCA |  |
|  | R | GTGAGCTGCATAACTTGAAGCG |  |
| c40199 | F | ATTCCTGGCTTCTGCTGCTC |  |
|  | R | GACCTCACCACCGTCCATTC |  |
| c35789 | F | TGGACCACCATGACACATTGT |  |
|  | R | GAACCTTCAACGCCTTCTTCAG |  |
| c39290 | F | ACAGAGCCGCCTTCAGGAT |  |
|  | R | GGCAACACGGTCTCAACACT |  |
| c42162 | F | GGAAGGAACGCAGTCTCTATTAC |  |
|  | R | AATACTCAGCCGATGCCTCAC |  |
| c43863 | F | GTCTCAGGAACTGCGAACTCA |  |
|  | R | TCTTCCACATTCCAGACATCACA |  |
| c42297 | F | CTCTCAATGGTGGCAAGTGC |  |
|  | R | CTTCCTGTTCCTGTGTTTGTCAC |  |
| c46798 | F | TCGCCACCTTCACCTCAAC |  |
|  | R | GGTAGCCGAATTGGGTCATCT |  |
| c38165 | F | CCGAGTCTGTGCTTGACCAA |  |
|  | R | GCTATTTCCCGACGCATCCA |  |
| c36175 | F | AGGATTCAAGCCGCCAACA |  |
|  | R | CCCCAAATGAGTCCGAGGA |  |
| c46612 | F | GTGCTCCTGATGGTAAATACGC |  |
|  | R | CCAGAACCAGAGTGCTGTCTT |  |
| c45307 | F | GATCACTCAGGACACACCATGT |  |
|  | R | GTCGTCTGCACCACAACTCTA |  |
| c48054 | F | ACAAACTTGGACAAGGTGGCT |  |
|  | R | TCTCTCTTCTTCATGGAAGCAAC |  |
| c30412 | F | CTCTCGGAGGACTTCAACGG |  |
|  | R | GTCAACCTGCTGTGGAATCG |  |
| c41666 | F | TCTCAACAGCCATTGACACCAC |  |
|  | R | GAGGTTTTGGTATCCACGTCCT |  |
| c47511 | F | GGGTTCATTATTGGAGTCACGC |  |
|  | R | CTTCGTCGCTTCCTCAATCTCT |  |
| c31798 | F | GGAGGTTGGACGCCGATCA |  |
|  | R | GGCTTCTCCCAAACGACGG |  |
| c31985 | F | GGGAAGAAGCAGCGAAACAG |  |
|  | R | AACCAACACGAGGAACTTCAGT |  |
| c43476 | F | GGTTTCAAAGAAGCAAAAGTGGC |  |
|  | R | GCCTTGAAATTGTATGACCCTGT |  |
| c44653 | F | CCATGGCTCACAACAACAACAC |  |
|  | R | ATCCCTCGGTTCTTCTTTGGTTT |  |
| c49683 | F | CCTGATCTGGCTCTAGGTTTGC |  |
|  | R | CAACAGAAATCCTCGTCCTCTCT |  |
| c38466 | F | GGACGACGACGACATTCACA |  |
|  | R | CGCCCAGTCTCTCACAACAA |  |
| c47334 | F | ATGGACGGCTATGATGCTGTT |  |
|  | R | GCCAATGCTGCTCTTATTGTCTG |  |
| c35494 | F | CGTGGCAGTGTTGGTTTCTC |  |
|  | R | TGGTAGTAACTGGCAATTTCTGC |  |
| c41475 | F | CCCACGTCACATACTAAGGTCAA |  |
|  | R | ATAGCGGAACGAGGCATAGC |  |
| c47088 | F | GGTATTTGCGTGCTCGGATTC |  |
|  | R | ACCACCACAGCGACTATGTC |  |
| c47127 | F | CTTGACTGTTTCTCTCGACGC |  |
|  | R | AGAGCACATGTAAGGCTCGTT |  |
| c23623 | F | AGCACTACTCTTCTTCTCCTCCT |  |
|  | R | CCACAGTACTCAGAAGTTCCATC |  |
| c36313 | F | CCAATAATCACAAGGCTCCAGTA |  |
|  | R | CTGTACTGTCAAGGCTTCCATAG |  |
| c40703 | F | AGTTGGAGATGTGCTAAGAGTGG |  |
|  | R | GATCGTTGTGGTGTTGGCATAG |  |
| c31769 | F | CTTCTCCAACTCCATCTCACCTT |  |
|  | R | GCCCTATGAGTGCCTCGTCT |  |
| c36895 | F | CCTCAATTCGGTAGCGGTTCC |  |
|  | R | AGCTGCTCCGTAGTTGTAGTTC |  |
| c46262 | F | TGTGCTGACGAATTAAGGACCAT |  |
|  | R | ATTCTCTCCCTCAAGCGTTTGT |  |
| c38775 | F | CGCTTCCATTCCTCTCATTGATC |  |
|  | R | GGTCGTCGGAGTAGTTCTTCAG |  |
| c43119 | F | CTCTGCACCACATCAGTCCTT |  |
|  | R | ATGCCTTCAACTCCTTCAACCT |  |
| c40192 | F | CTGAGCAGTACCATCCGAGTT |  |
|  | R | GTGGAGGCAGGAACTTGGAA |  |
| c37744 | F | TCCAATCCCATCGTCCATTCC |  |
|  | R | CGTCCATTGCTGTTGCTCTG |  |
| c42874 | F | TGCCGTCCAAGTCCAACAAG |  |
|  | R | CCTCCGACAGCTTCTGTAACTC |  |
| c34352 | F | GGCATAATCGACCAGGCTGAT |  |
|  | R | TGTCTCGTCACAGTAGTCACCT |  |
| c36015 | F | CCTTCCAATGCTGATCGTTCG |  |
|  | R | GTAGCAATCCAAATTGTCTCCGTAG |  |
| c42529 | F | GCTTATTGTTCTACTTGGGATGCTG |  |
|  | R | CTTGTTGATAACCTCTTCCATCGG |  |
| c36895 | F | CAGACACACATTTTGGAGAAGATATC | Cloning |
|  | R | AATTGAGACAGAATTTGACTCCAACAC |  |
| c42297 | F | GTTCCCCGCCACCAATCTCA |  |
|  | R | AAACGGGCCTCATATTTTCCAAT |  |
| c46798 | F | CGGTGGAGTAGTTGAGTGGGTTAC |  |
|  | R | TCAAGCCCCACAATTGAAAGACG |  |
| c46612 | F | ATCAACAAAACAACCACAACACATC |  |
|  | R | CAAGAGTAGTCAAAACGTTGAATTTCATTG |  |
| c36015 | F | GTTGGAAAAACGAGGAGAGAACATC |  |
|  | R | AATAAGCAAAATATTATTAGAAAGATAAGG |  |
| c42529 | F | TATTGCAACAACGTGGACGCTC |  |
|  | R | AACGCCAAAAACTCCATATCATGTTAC |  |
| c34352 | F | TTTATAATTCCACTTACATCTATTTCAC |  |
|  | R | TTAATTTCTTGTTTCTAAGGTTAAGC |  |
| MlChiIA | F | ggatccGAGCAATGTGGTCAGCAAG | Prokaryotic Expression |
|  | R | aagcttTTACATAGCATCCACCATGAGTCC |  |
| MlChiIB | F | ggatccCAAAACTGCGGGTGTGC |  |
|  | R | aagcttCTAGCAAGTAAGGTTTTCGCCA |  |
| MlChiIC1/2 | F | ggatccTTTGGAGATGTTAGAAGCA |  |
|  | R | aagcttCTACTGATTAAACGGACGCTG |  |
| MlChiID1/2 | F | ggatccGAACAATGTGGTAGGCAAGTCG |  |
|  | R | aagcttTTATTGTTCATCAACAATAGAAAGAAG |  |
| MlChiV1-1/2 | F | ggatccCAATCCACGGTGAAAGCCG |  |
|  | R | gcggccgcCTAGYTCGTCGATCTCCATTC |  |
| MlChiV2-1/2 | F | ggatccCAATCCACGGTGAAAGCCG |  |
|  | R | gcggccgcTCAAGCCCCACAATTGAAAGAC |  |
| MlChiIV1/2/3 | F | gagctcCAAAATTGTGGGTGCAGCTC |  |
| MlHEL1/2 | R  F  R | aagcttTTAGCAAGTGAGATTGGATCCAGG  ggatccCAGAGTGCGACTAATGTGAG  aagcttTTAGTCACCACAGTTGACGAACTC |  |
