# Supplementary material for: Transcriptomic Analysis Reveals Candidate Genes Responsive to Sclerotinia scleroterum and Cloning of the Ss-Inducible Chitinase Genes in Morus laevigata
Source: Int J Mol Sci. 2020 Nov 7;21(21):8358. doi: 10.3390/ijms21218358 (PMC7664649; doi:10.3390/ijms21218358)
Supplement: Supplementary file 1 [file ijms-21-08358-s001.zip › Supplementary Figure.docx]

c

**Figure S1**. Expression patterns of the 42 candidate genes in different organs of *M. laevigata.* All data were shown as the mean +SE (n = 3). Ro:Root, Le:leaf, St: Stem, Fl: Flower, Fr: Fruit

**Figure S2.** Expression patterns of the 42 candidate genes in mixed-sample with *S. sclerotinrum* induction of three mulberry cultivars. All data were shown as the mean +SE (n = 3).

**Figure S3.** Expression patterns of the 42 candidate genes with *S. sclerotinrum* induction at different stages in three mulberry cultivars. All data were shown as the mean +SE (n = 3).


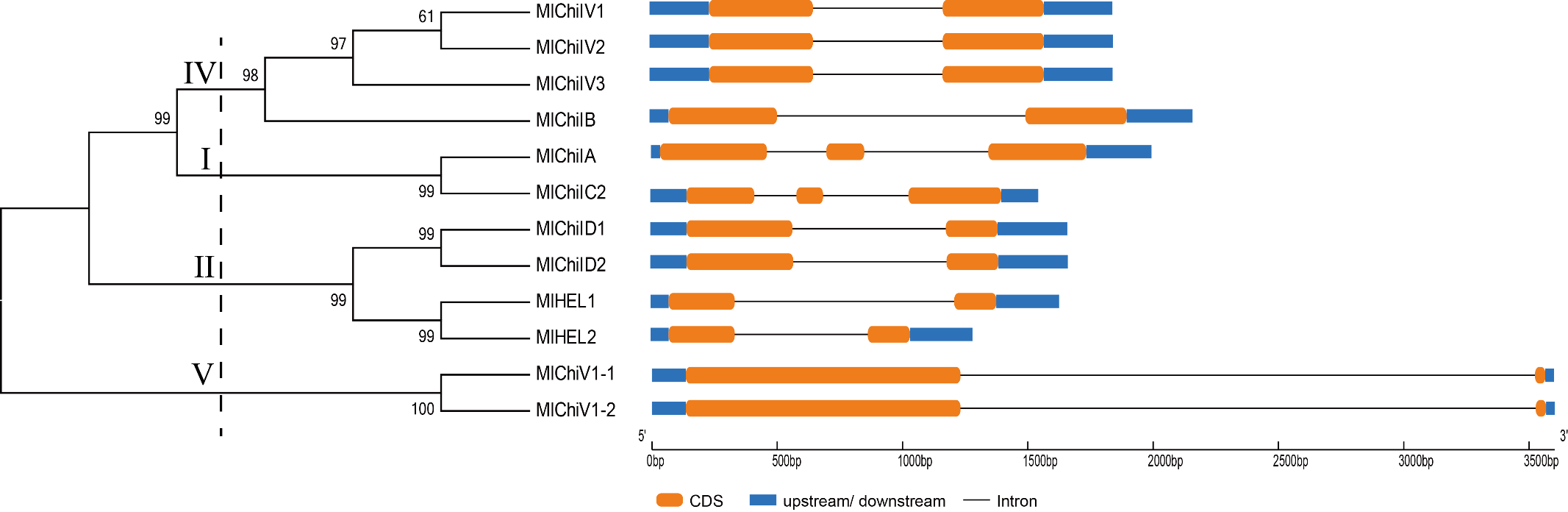


**Figure S4.** Phylogenetic and gene structure analysis of the chitinase genes in *Morus laevigata*. The un-rooted NJ-tree was constructed using the full-length protein sequences of the nine chitinases with 1000 bootstrap replicates in MEGA X. The exons and introns were annotated by sequence alignment of the gDNA and full-length ORF sequences of each gene.


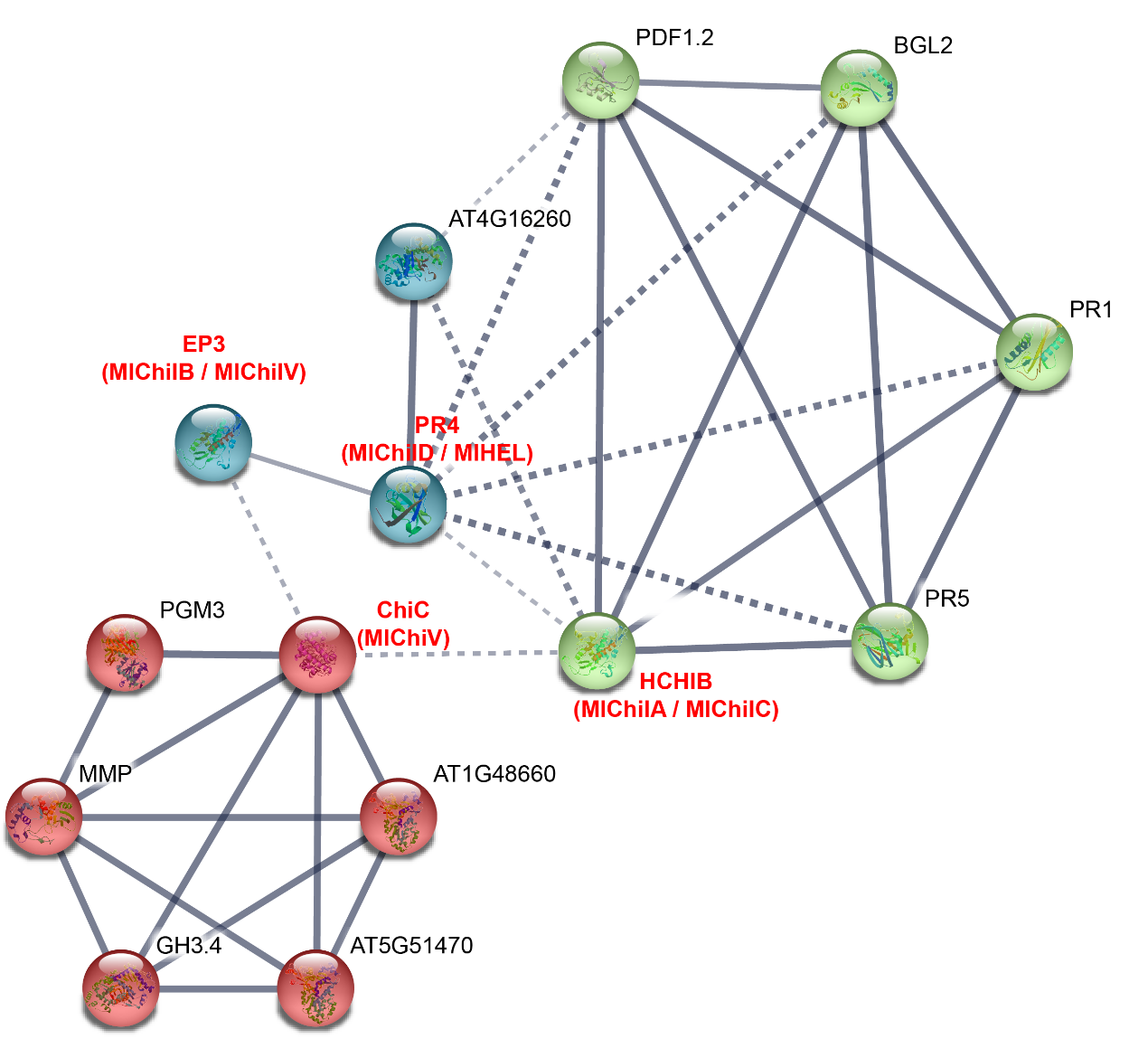


**Figure S5.** Interaction network of the chitinase proteins in *Morus laevigata* according to the orthologs in *Arabidopsis thaliana.* The network type was set as full network, the network edges were represented as confidence and the line thickness indicated the strength of data support, the network clustering method was kmeans and the clustering number was set as 3, and other parameters were default.

*

*

**Figure S6.** SDS-PAGE analysis of the expression of recombinant proteins in *E.coli* BL21. (A)The expression of the IPTG induced and uninduced recombinant proteins. (B) The expression of the IPTG induced recombinant proteins in the supernatant and precipitation of the bacteria solution. M: marker, EV: empty vector, U: uninduced, I: induced, S: supernatant, P: precipitation.
